# A Prevalent Habit Condemned

**Published:** 1920-04-17

**Authors:** 


### INFECTIOUS DISEASE IN CHILDREN.

### A Prevalent Habit Condemned.

After eighteen months of exhortation to bear in mind at least the essentials of disease transmission through the agencies coughing, sneezing, and other physiological urgencies which may arise in the human body, it seems likely that the public has by now come thoroughly to understand that these things concern not only the individual reputation for "manners," but both the individual and collective health of the people as well. Most of the possible ways in which disease may be disseminated by direct or indirect contact, by the diffusion of atmosphere-carried fluid droplets, and the increased possibilities of all in overcrowded communities or buildings are by now so firmly insisted upon in all health propaganda that we need not here waste the reader's time by repeating them.

There is, however, one subject which, perhaps from its universal and accepted prevalence, alone has largely escaped this wholesale denunciation. This habit is the persistent kissing of children by admiring relatives and friends. An increasing number of people are beginning to understand the risk that the child trained to kiss the friendly stranger or affectionate relative may run, and, at the risk of a somewhat undemonstrative generation—according to recent standards, at least—are encouraging their children to refuse point blank to kiss anyone. And surely a gesture from a dainty little hand can express far more than the most resounding kiss; but here it is no part of our intention to enter into this part of the discussion. The point which matters is the fact that disease is transmitted with unbelievable frequency in this apparently harmless way, and our attention is drawn to a number of instances collected by speakers at the last meeting of the American Pediatric Society. Two other things there are which must be remembered—namely, the far greater susceptibility of the child to infectious disease, and also the fact that, once infected, the child is so superlatively liable to develop complications which may wreck its whole future life.

Let us, therefore, take the instance of a case investigated by Dr. Shaw, of Albany, and reported in the *Medical Record* of New York. Medically there is to the doctor but little novelty in the occurrence described, but if these words should be honoured with the attention of any who could directly or indirectly work to bring about a general suppression of the kissing habit, this specific instance may aid them to the decision that such a movement is well worth while.

Dr. Shaw's investigation was conducted primarily to trace the cause of a sporadic case of cerebro-spinal meningitis in a baby five months old, breast fed, and living at a farmhouse. All who came to the house during the previous week could be found and examined because of the isolated position of the farm; no former cases were recorded in this place. The baby had been kissed and fondled by a demobilised soldier who had returned from France that month. The baby was taken ill a few days afterwards with typical cerebro-spinal meningitis. The soldier stated that as far as he knew he had never seen, been in contact with, or heard of a case of meningitis in his regiment, but he was found bacteriologically to be a meningo-coccus carrier. All other visitors to the farm gave negative nasal cultures, and the chain was completed by the discovery that the meningococci obtained from the soldier were agglutinated by the cerebro-spinal fluid obtained from the baby.

If we enlarge on the possibilities arising from the presence of this soldier carrier in a community more crowded and with a number of youthful infant relations of relatively high susceptibility, a glimpse might be obtained of the possible spread of disease in this way. It is our duty to keep abreast of public health progress and assist in educating the public towards a new order of things which better medical knowledge has brought into being. From our quoted instance many lessons may be derived. Not only the dangers which surround children in their close contact with older carriers, but the value of the whole doctrine of carrier tracing and isolation. The one aspect sufficient for our immediate purpose, and may well be used to illustrate the dangers coincident with a habit we must most heartily condemn.

